# Supplementary material for: Identifying and prioritizing potential human-infecting viruses from their genome sequences
Source: PLoS Biol. 2021 Sep 28;19(9):e3001390. doi: 10.1371/journal.pbio.3001390 (PMC8478193; doi:10.1371/journal.pbio.3001390)
Supplement: S1 Text — Extended discussions of the viral genome features identified by our model as important for prediction of human infection status and potential biological mechanisms underlying the shape of their relationships with human infection status. (PDF) [file pbio.3001390.s001.pdf]

# Identifying and prioritizing potential human-infecting viruses from their genome sequences: S1 Text

Nardus Mollentze, Simon Babayan & Daniel Streicker

## Viral genome compositional predictors of human infection

Our results showed that combining information on a variety of genome compositional biases can differentiate viruses known to infect humans from those which have not been reported to do so. Moreover, the aspects of genome composition which predicted human infection in some cases did so across divergent viruses (e.g., from different taxonomic classes), signalling potential for convergent evolutionary processes which predispose the capability for human infection through mechanisms of virus-host adaptation which are not currently understood. We therefore carried out additional analyses exploring the influence of our input genome composition measures on model predictions in order to aid future research using experimental approaches to manipulate viral genome composition.

First, we compared the frequency at which features calculated directly from the viral genome or in reference to three sets of human transcripts were included in the final model. We hypothesized that human similarity features would be more prominent if viral mimicry of host transcripts increased viral fitness in humans (either due to common selective pressures from nucleotide sensing defences or enhanced translation) or if dissimilarity to human transcripts was somehow adaptive. In contrast, unreferenced viral genomic features might be more prominent if specific aspects of genome composition were adaptive for viruses, but neutral for human genes. Among the 125 features retained in the final model, most described similarity of viral genomic features to the transcripts of human genes (ISGs = 27.3%; housekeeping genes = 22%; remaining genes = 12.7%). The remaining 38% of features described viral genomic composition directly. Although this excess frequency of human similarity-based features among retained features is partly explained by the larger number of such features available, compositional similarity to housekeeping genes and ISGs also tended to have a larger magnitude of influence on predictions than unreferenced viral genomic features (Fig 2B). Moreover, when different representations of the same genome composition measure were retained in the final model (N = 20 compositional measures, 50 features), the human similarity version tended to be more important than the unreferenced measure of viral genome composition (Fig 2C). These results imply that when retained, features that described similarity to human genes had more powerful effects on model predictions of human infection.

Since many genome compositional features were correlated within viral genomes, we further summarised the total predictive power of 31 discrete clusters of features identified by affinity propagation clustering of pairwise Spearman correlations between observed feature values (Fig 2D & S9–S10 Figs). As expected, many clusters (41.9%) contained a mixture of different feature types (amino acid biases, codon biases, and dinucleotide biases), with 4 clusters containing combinations of all three. As a result, no single genome composition measure could be isolated as the driving force behind the ability of models to predict human infection ability. Most clusters also contained both unreferenced viral genomic and similarity-based features (Fig 2D), but ISG similarity features were identified as the exemplars (i.e., the most central or representative datapoint) of the largest number of clusters consisting of ≥ 3 features (36%, observed/expected ratio [OER] = 1.40). Housekeeping and remaining gene similarity features were identified as the exemplars of 24% and 16% of such clusters, respectively (OER = 1.08 and 1.64), leaving unreferenced genome features under-represented

as exemplars (24%, OER = 0.57). Measures of similarity to human genes were therefore the best representation of the majority of feature clusters predicting human infection ability (Fig 2D & S9 Fig).

Acknowledging the challenge in interpreting correlated genomic features in isolation, we next explored the shape of relationships between the values of each feature and the predicted log odds of human infection (S10 Fig). For human similarity features, we observed patterns consistent with both compositional mimicry and, more rarely, compositional distancing. For both positive and negative effects, patterns could be linear or non-linear (e.g., extreme values increasing the odds of human infection, with all others being neutral or negative; see for example similarity in TpC-dinucleotide bias at codon bridges [brTpC similarity]). Similarly, for unreferenced viral genomic features, we observe both positive (e.g., GpT-dinucleotide bias and serine amino acid bias) and negative (e.g., leucine amino acid bias and GpA dinucleotide bias at codon bridges) relationships with human infection, implying that both avoidance and favouring of certain genomic features can increase the odds of human infection. This finding in part arises because many of the genome compositional features we included are (or form part of) redundancies in the genetic code, such that bias against one feature necessarily implies bias for another, along with uncertainty surrounding which parts of the human genome might be mimicked. For example, high levels of brTpC similarity to remaining genes was associated with increased likelihood of infecting humans, but high levels of similarity in the same feature to the transcripts of housekeeping genes had the opposite effect (S10 Fig). However, these two measures of similarity were correlated (Spearman correlation = 0.53) and were grouped in the same cluster of features, with brTpC similarity to the transcripts of remaining genes as the exemplar (cluster 2, S9 Fig). The transcripts of remaining genes tended to have a slightly lower TpC bias at codon bridges (median = 0.95) than those of housekeeping genes (median = 1.03). Together, these observations suggest that if mimicry of bridge TpC bias is the factor under selection (as opposed to any of the other correlated features in cluster 2) – it appears that matching remaining genes is more important, while the measured similarity to housekeeping genes may simply act as a marker for too-high TpC content. Similar patterns were observed across the majority of clusters, with at least one of the correlated features in each of these clusters supporting a role for mimicry of human gene transcripts. However, three clusters stood out as pointing to a negative relationship between similarity and human infection ability without any correlated features supporting a role for mimicry (clusters 14, 20, and 26 in S9 Fig). These apparent compositional distancing effects tended to have weak effects compared to compositional mimicry features and the mechanisms driving their association with human infection are unknown. It is possible that distancing reflects complex trade-offs between different features or mimicry of a set of human gene transcripts that is poorly captured by the three gene sets summarised here. Alternatively, apparent compositional distancing may arise from selection to avoid specific features at all costs (e.g. those targeted by antiviral defences), where a low occurrence of the feature may be more important than matching the particular frequency found in human genes subject to additional constraints. Interestingly, several features strongly linked to human infection here were also identified among the most powerful features for discriminating reservoir hosts in a smaller dataset of RNA viruses, including leucine and serine amino acid bias and GpT-dinucleotide bias, supporting their potential role in influencing viral host range (see figure S10 in [1]).

Taken together, these results suggest that matching (or, potentially, diverging from) human genes either serendipitously or by selection in natural host species, along with other mechanisms by which viruses adapt their genome composition in ways that are independent of host genome composition, predictably predisposes some viruses to be capable of infecting humans. That the viruses studied here exploit a diversity of approaches is intuitive since our

dataset contained a diverse array of RNA and DNA viruses which are likely both to face different selective pressures and to employ different solutions in the case of common selective pressures. Even within a single virus species, a combination of effects which involve, for example, avoidance of innate immunity by reducing certain dinucleotide motifs and mimicry of other motifs which enhance translation could together enhance the likelihood of human infection. Although the exact mechanisms by which genome compositional biases affect viral fitness are only beginning to be understood, the existence of these fitness effects, including effects on host range, are increasingly evident for a variety of viruses [2–5]. Experimental approaches involving viral genome re-coding are well developed from the virus attenuation literature [reviewed in 6] and would be useful to isolate the effects of individual aspects of genome composition suggested by our predictive model.

**References**

1. Babayan SA, Orton RJ, Streicker DG. Predicting reservoir hosts and arthropod vectors from evolutionary signatures in RNA virus genomes. Science. 2018;362: 577–580. doi:10.1126/science.aap9072

2. Coleman JR, Papamichail D, Skiena S, Futcher B, Wimmer E, Mueller S. Virus Attenuation by Genome-Scale Changes in Codon Pair Bias. Science. 2008;320: 1784–1787. doi:10.1126/science.1155761

3. Mueller S, Coleman JR, Papamichail D, Ward CB, Nimnual A, Futcher B, et al. Live attenuated influenza virus vaccines by computer-aided rational design. Nature Biotechnology. 2010;28: 723–726. doi:10.1038/nbt.1636

4. Martrus G, Nevot M, Andres C, Clotet B, Martinez MA. Changes in codon-pair bias of human immunodeficiency virus type 1 have profound effects on virus replication in cell culture. Retrovirology. 2013;10: 78. doi:10.1186/1742-4690-10-78

5. Shen SH, Stauft CB, Gorbatsevych O, Song Y, Ward CB, Yurovsky A, et al. Large-scale recoding of an arbovirus genome to rebalance its insect versus mammalian preference. PNAS. 2015;112: 4749–4754. doi:10.1073/pnas.1502864112

6. Martínez MA, Jordan-Paiz A, Franco S, Nevot M. Synonymous Virus Genome Recoding as a Tool to Impact Viral Fitness. Trends in Microbiology. 2016;24: 134–147. doi:10.1016/j.tim.2015.11.002
