# Supplementary figures and images for: Identifying and prioritizing potential human-infecting viruses from their genome sequences
Source: PLoS Biol. 2021 Sep 28;19(9):e3001390. doi: 10.1371/journal.pbio.3001390 (PMC8478193; doi:10.1371/journal.pbio.3001390)

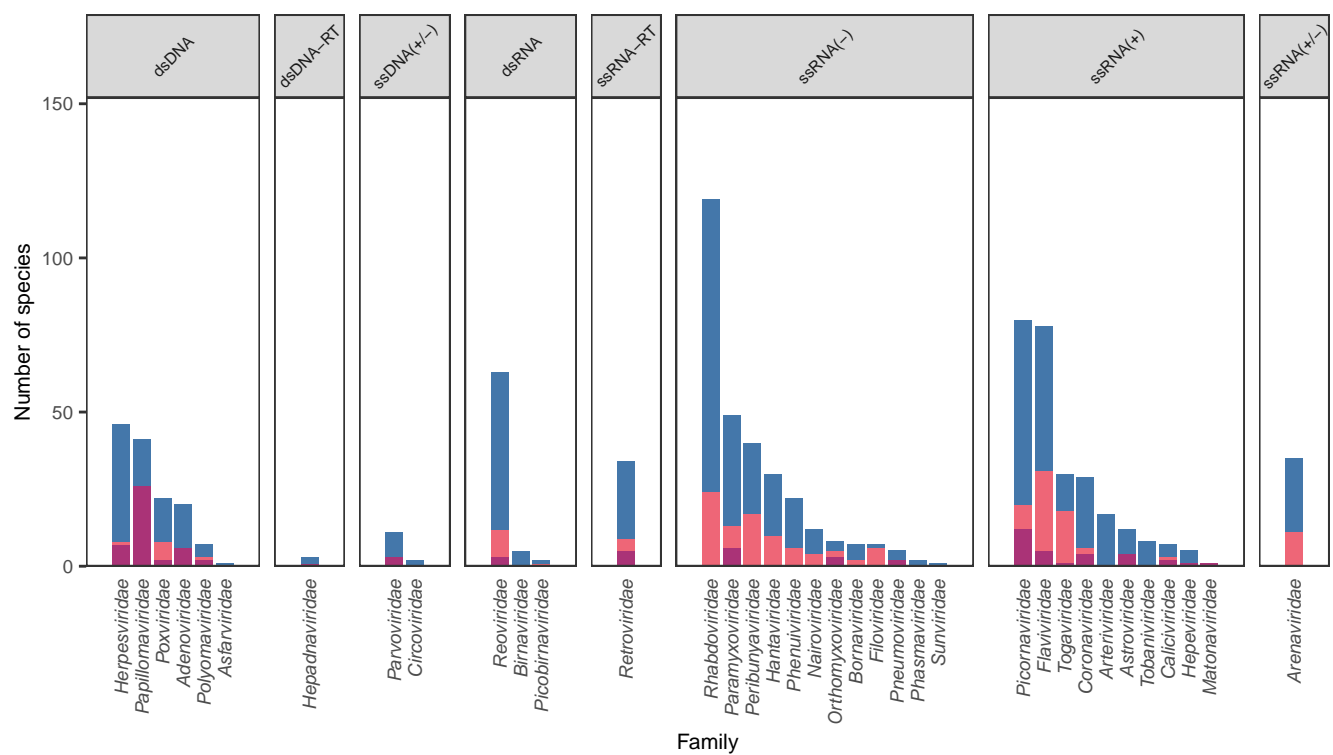

Supplement: S1 Fig — Viruses (N = 861 species) predominately transmitted among humans (purple) or from animals to humans (zoonoses, pink) were combined to form the positive class of human-infecting viruses. All other virus species, for which no human infections have been detected (blue), were used as the negative class when training models. Numerical data underlying this figure can be found at https://github.com/nardus/zoonotic_rank/tree/main/FigureData (doi: 10.5281/zenodo.4271479). (PDF) [file pbio.3001390.s003.pdf]

**A**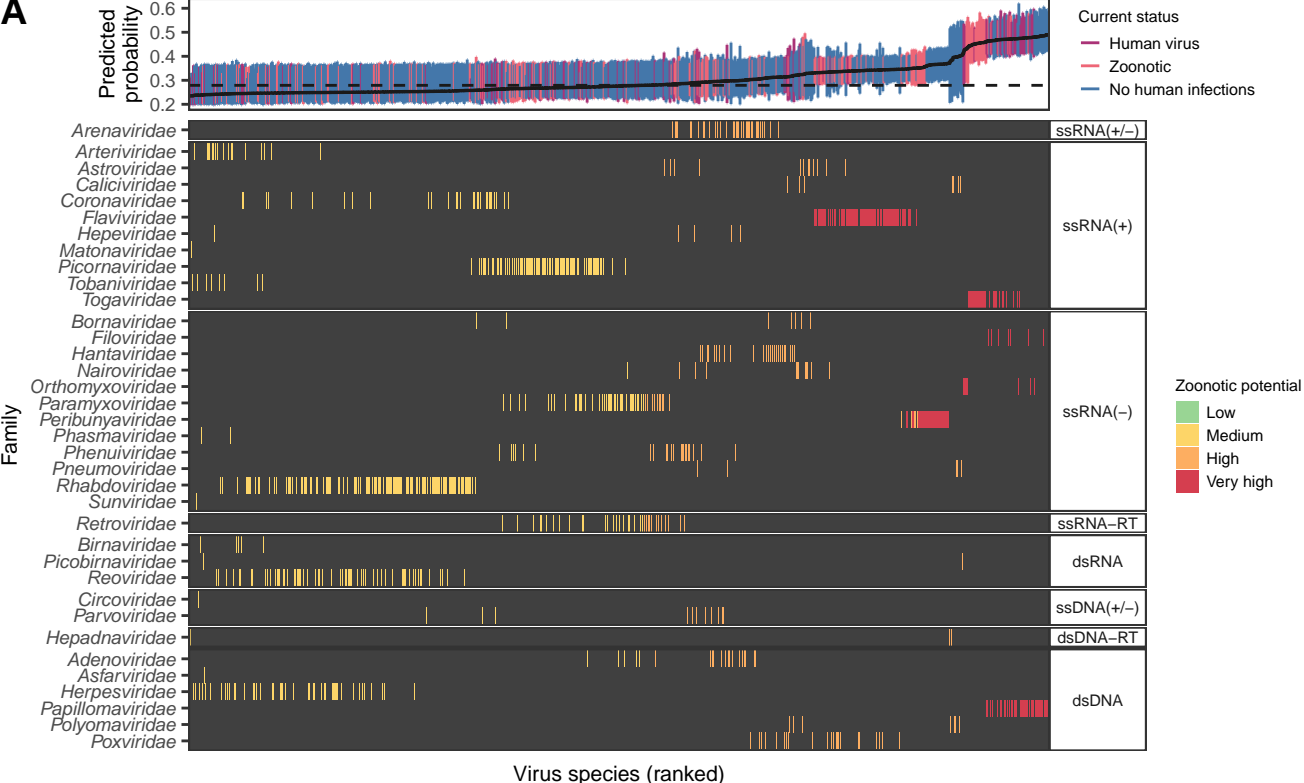**B**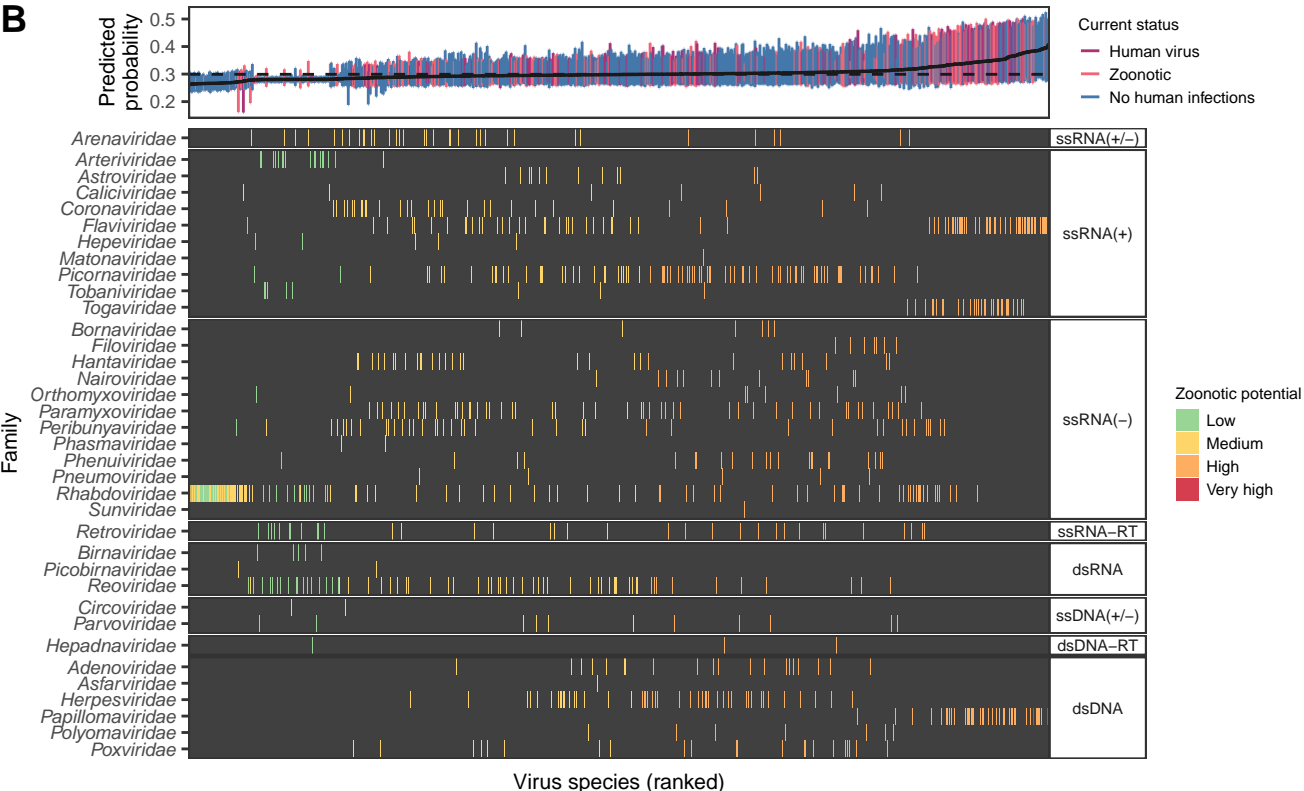

Supplement: S2 Fig — Viruses from the training data are shown ranked by their predicted probability of infecting humans produced by bagged versions of (A) the taxonomic feature set-based model and (B) the PN-based model. In the top panel of each plot, a solid gray line shows mean bagged probabilities, while colored error bars highlight the region containing 95% of predictions from the iterations used in bagging. A dashed line shows the cutoff that balances sensitivity and specificity. The lower panel in each plot highlights the location of viruses from different families (colored bars) and contains a dark gray background for clarity. Clustering of zoonotic risk by virus family in the taxonomic feature set-based model (A, lower panel) shows that this model is unable to discriminate high and low risk viral taxa within viral families. This was expected, since this model contained only features resolved at the family level or above. A sequence similarity-based approach was expected to perform better, but while the PN model (B) does show improved resolution, it still fails to separate risk within certain viral families. For example, papillomaviruses and most flaviruses are ranked as high risk, despite the presence of nonzoonotic species within each family. In contrast, the genome composition model was considerably more accurate (Fig 1, S3 Fig) and was able to assign viruses from the same family into a broader range of risk categories (see S4 Fig), consistent with our current biological understanding that zoonotic ability varies within viral families. Numerical data underlying this figure can be found at https://github.com/nardus/zoonotic_rank/tree/main/FigureData (doi: 10.5281/zenodo.4271479). PN, phylogenetic neighborhood. (PDF) [file pbio.3001390.s004.pdf]

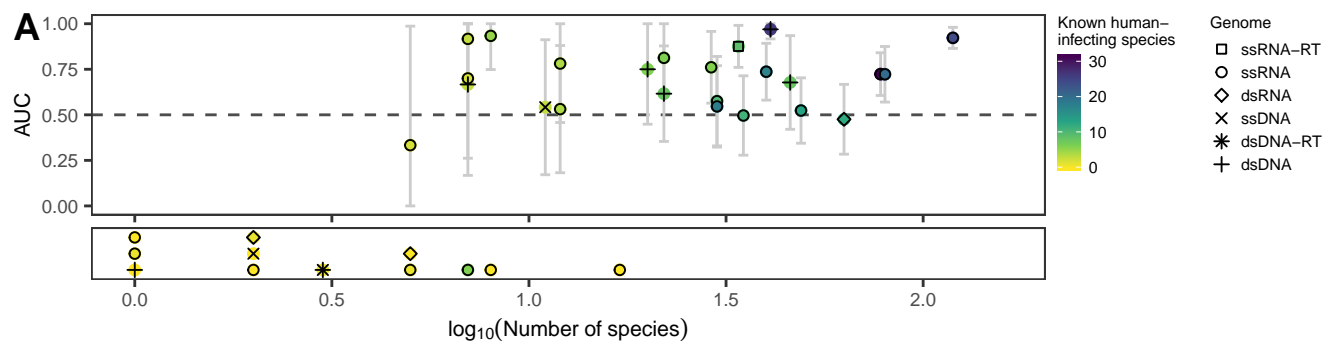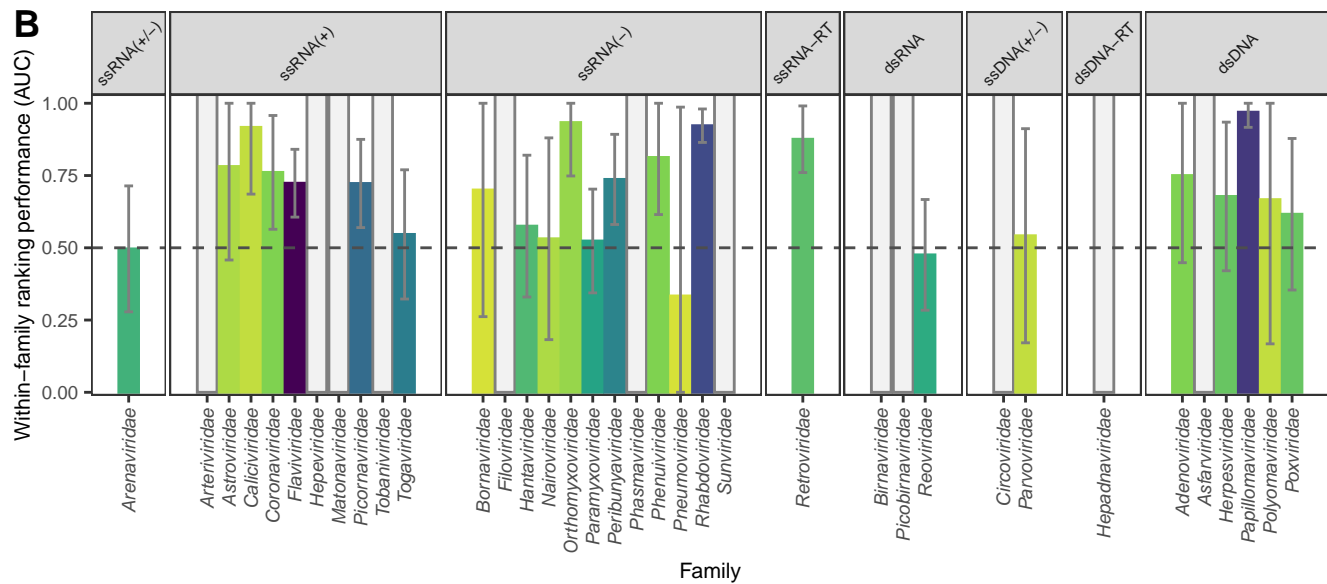

Supplement: S3 Fig — AUC here measures the probability of accurately ranking known human-infecting viruses above other viruses from the same family, when ranking viruses using the output from the bagged model based on all genome feature sets. AUC values could not be calculated for families containing <2 human-infecting viruses or <2 viruses not known to infect humans. These families are illustrated in the lower plot in (A), where the y-axis is unitless and overlapping points are stacked, and as gray bars in (B). CIs were calculated using the method of [47,48] and highlight the difficulty of assessing within-family AUC given the relatively small numbers of viruses currently known in most families. We detected no obvious taxonomic pattern in the variation of within-family AUC values. Numerical data underlying this figure can be found at https://github.com/nardus/zoonotic_rank/tree/main/FigureData (doi: 10.5281/zenodo.4271479). AUC, area under the receiver operating characteristic curve; CI, confidence interval. (PDF) [file pbio.3001390.s005.pdf]

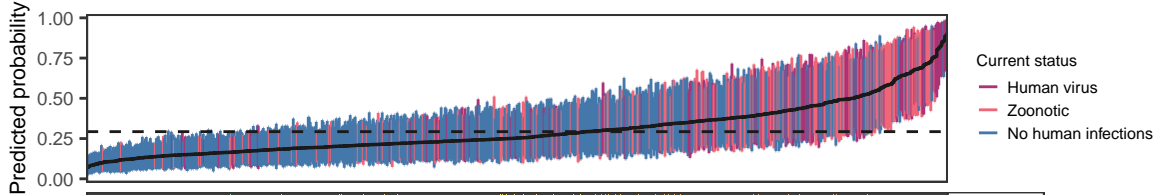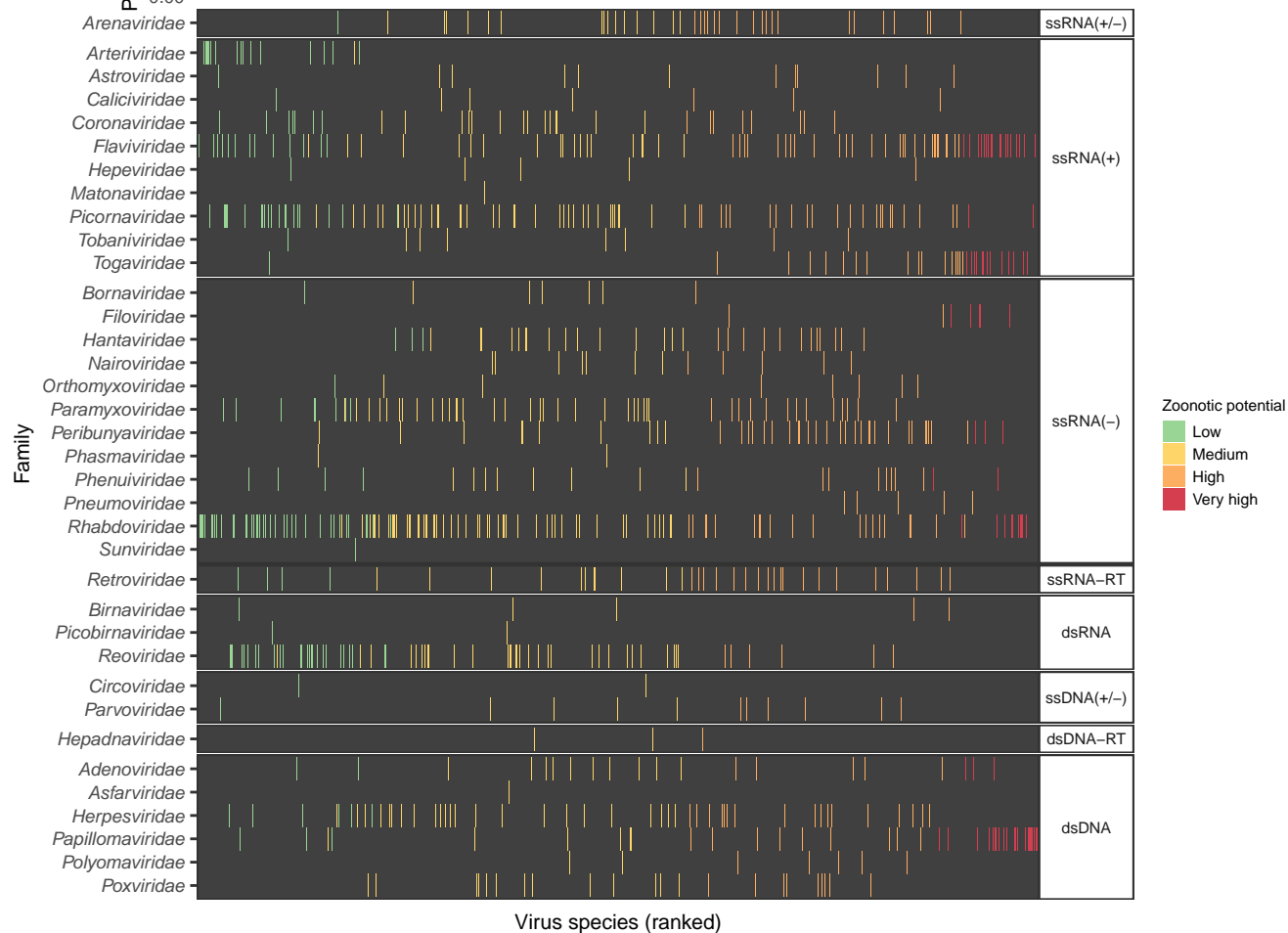

Supplement: S4 Fig — Viruses in the training data are shown ranked by their mean predicted probability of infecting humans, produced by bagging across iterations of the combined genome feature–based model. In the top panel, a solid gray line shows mean bagged probabilities, while colored error bars highlight the region containing 95% of predictions from the iterations used in bagging. A dashed line shows the cutoff that balances sensitivity and specificity. The lower panel highlights the location of viruses from each family using colored bars and contains a dark gray background for clarity. For a detailed list of viruses and their ranks and priorities, see S1 Table. Model predictions within viral families span risk categories, illustrating the power to discriminate risk at higher taxonomic resolution than models based on conserved features of virus biology (e.g., ability to replicate in cytoplasm or be transmitted by arthropod vectors) or alternative models based on taxonomy or PN (S2 Fig). For the numerical data underlying this figure, see S1 Table. PN, phylogenetic neighborhood. (PDF) [file pbio.3001390.s006.pdf]

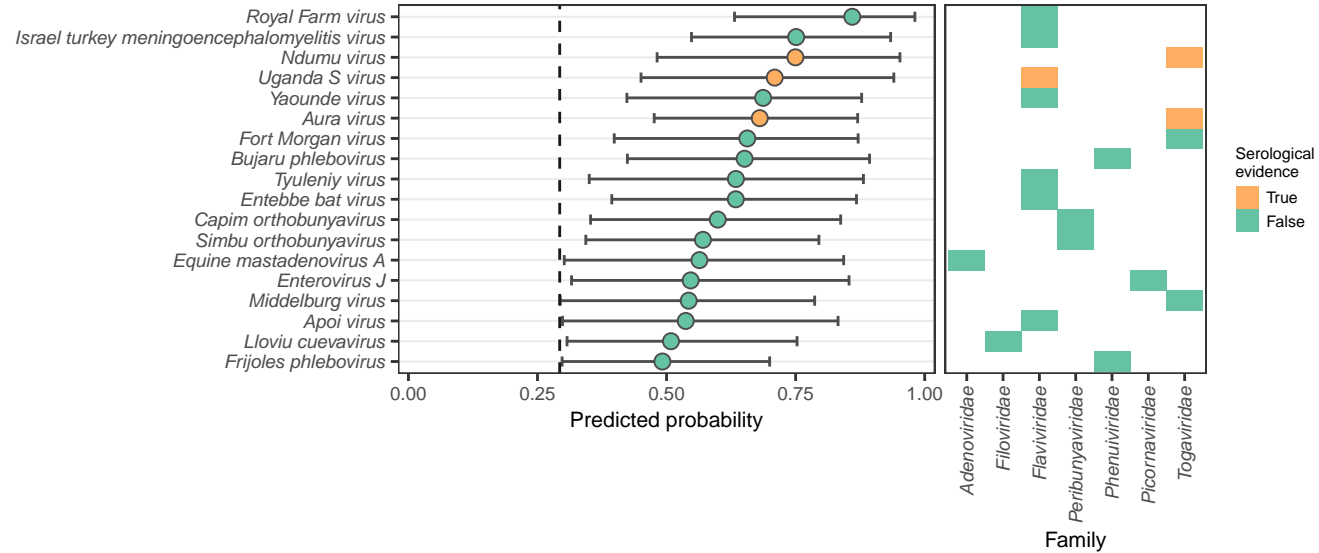

Supplement: S5 Fig — Points and CIs in the left panel show the bagged mean and 95% confidence range on the predicted probability of human infection when using the combined genome feature–based model. Each virus species shown was included in the training data as not currently known to infect humans, but was nevertheless classified in the “very high zoonotic potential” category when included in test sets, which means ≥95% of the bagged iterations predict these viruses as human infecting. The right panel shows evidence of serological infection for 3 of these viruses (orange), from [5,17]. Absence of serological evidence may reflect lack of diagnostic testing, lack of quantifiable antibody responses, lack of human exposures, or inability to infect humans. For the numerical data underlying this figure, see S1 Table. CI, confidence interval. (PDF) [file pbio.3001390.s007.pdf]

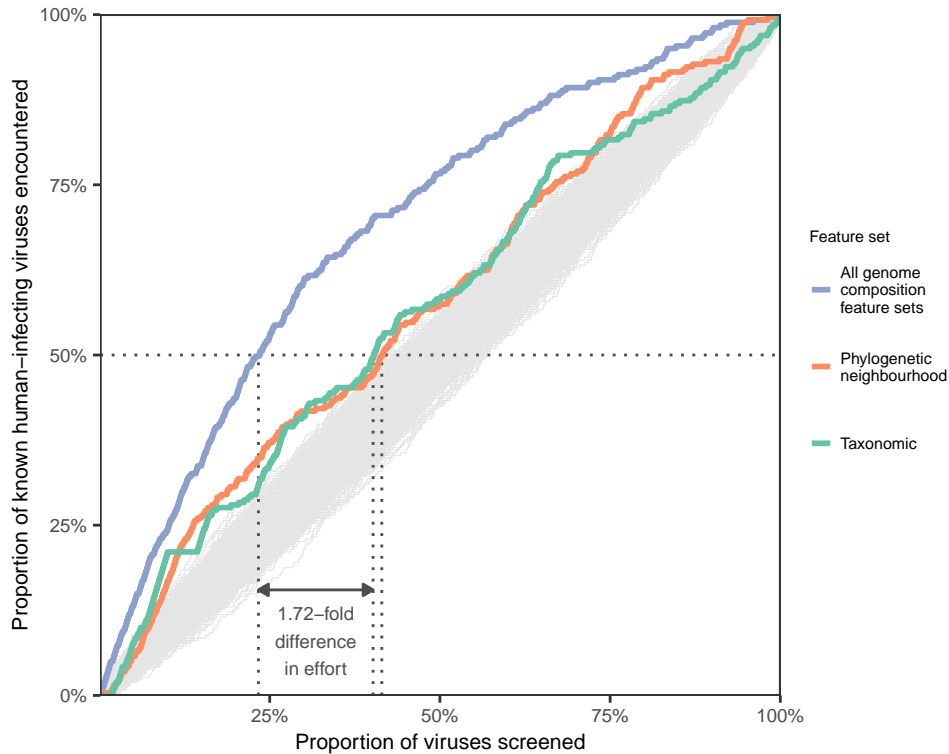

Supplement: S6 Fig — For each feature set (or combination of feature sets), viruses were ranked based on bagged predictions across the top 100 out of 1,000 training iterations. Dotted lines highlight the proportion of all viruses in the training and evaluation data that need to be screened to detect 50% of known human-infecting viruses. Gray lines show the range of accumulation curves expected from random screening in a dataset of this size, simulated by randomly shuffling viruses 1,000 times. Numerical data underlying this figure can be found at https://github.com/nardus/zoonotic_rank/tree/main/FigureData (doi: 10.5281/zenodo.4271479). (PDF) [file pbio.3001390.s008.pdf]

**A**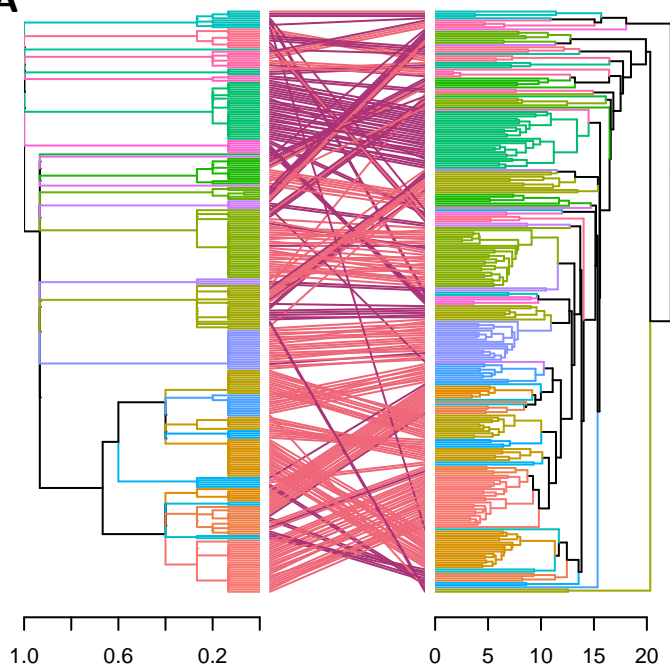**B**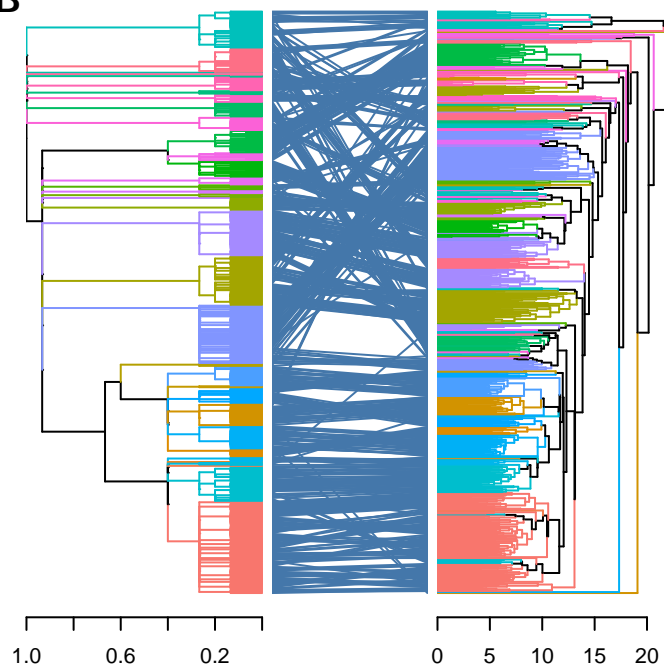**C**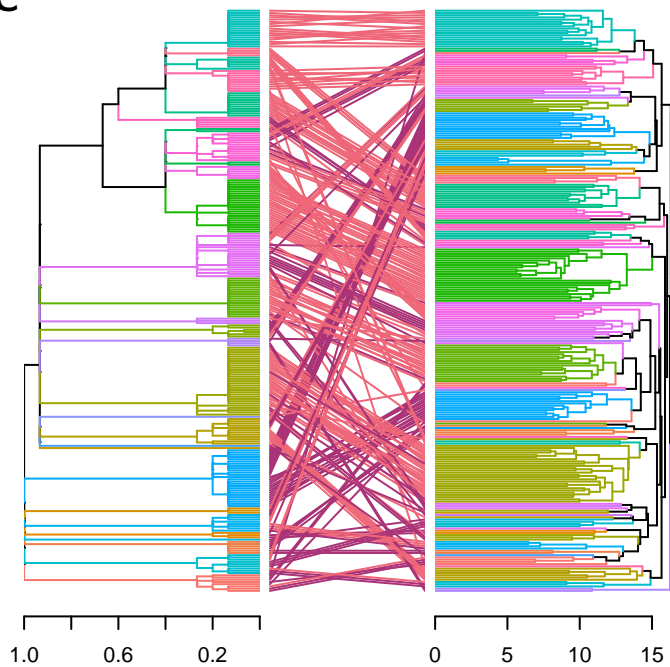**D**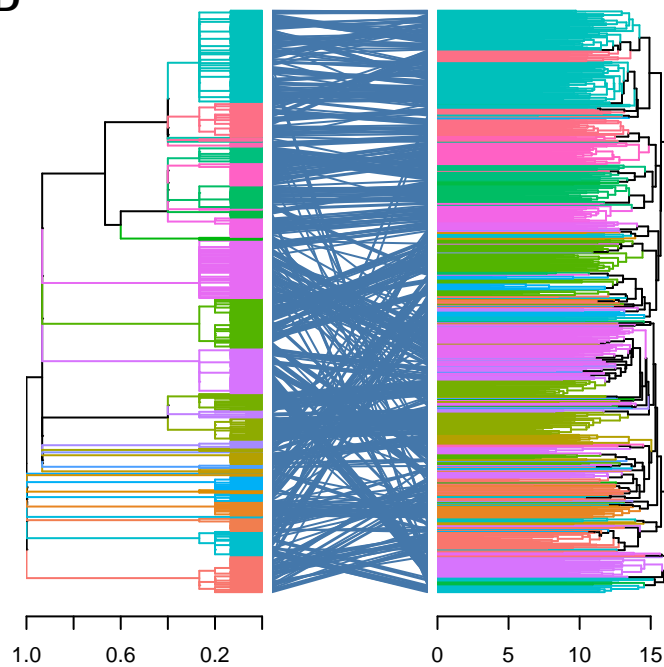

Supplement: S7 Fig — Tanglegrams compare hierarchical clustering of viruses by taxonomy (left in all panels) to clustering by (A and B) genome features or (C and D) model explanations from the combined genome feature–based model (SHAP values). Lines connect individual species in each dendrogram. Terminal branches are colored by family, while connecting lines are colored to indicate human-transmitted (purple), zoonotic (pink), and other viruses (blue). Note that the colors assigned to each family depend on the order of families in the dendrogram and have been optimized for distinguishability of neighboring families, meaning colors do not match across panels. Numerical values used for clustering are displayed in S10 Fig, and can be found at https://github.com/nardus/zoonotic_rank/tree/main/FigureData (doi: 10.5281/zenodo.4271479). SHAP, SHapley Additive exPlanations. (PDF) [file pbio.3001390.s009.pdf]

⊕ Null distribution ⊕ Observed ( $q < 0.05$ ) ⊕ Observed ( $q \geq 0.05$ )

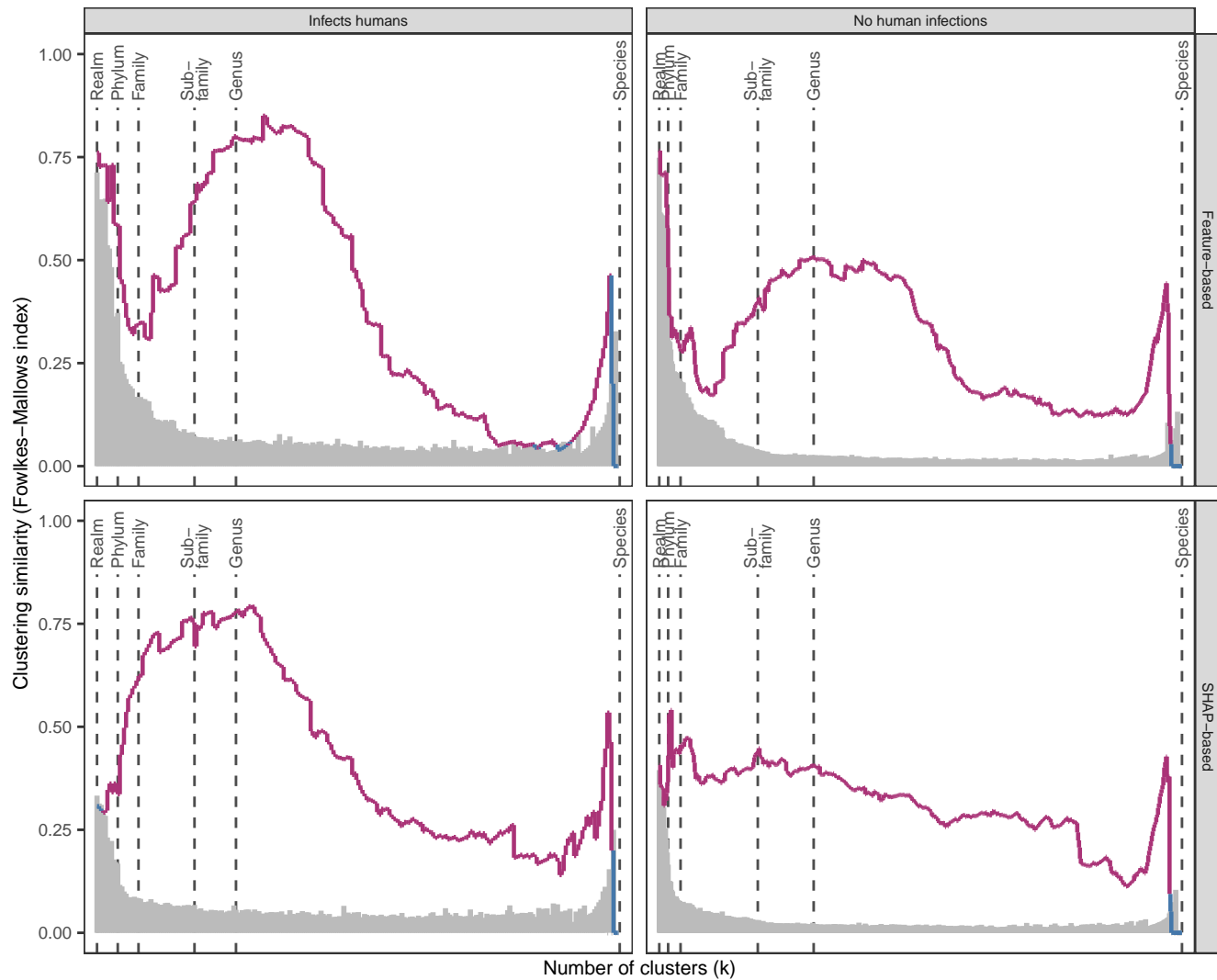

Supplement: S8 Fig — Each panel measures clustering similarity (purple/blue line) when cutting virus taxonomy and either feature-based (top row) or SHAP value-based dendrograms (bottom row) into different numbers of clusters. Increasing the number of clusters (k) therefore compares clustering at increasingly shallower parts of the respective dendrograms (i.e., comparing more closely related viruses). A subset of taxonomic levels corresponding to different levels of subdivision is labeled for orientation. A clustering similarity of 1 would indicate complete agreement in the membership of all clusters, while a similarity of 0 indicates no agreement [46]. In all panels, empirical null distributions (gray) were obtained by randomly shuffling the labels of both dendrograms 1,000 times. Clustering similarities significant at the 0.05 level (after Bonferroni correction) are illustrated in purple, while values that are not statistically distinguishable from the empirical null distribution are shown in blue. The correspondence between all dendrograms and virus taxonomy was generally higher than expected by chance. However, clustering of human-infecting viruses shows a lack of correspondence between how genome features are used in the model (as measured using SHAP values, bottom row) and the highest levels of virus taxonomy, even when such information was available among the input features (top row). Specifically, for both human-infecting and nonhuman-infecting viruses, correspondence between clustering and virus taxonomy declines from the family to the realm levels in SHAP-based clustering, but increases in genome feature–based clustering. This indicates that while genome feature–based clustering closely mirrors high-level virus taxonomy, SHAP-based clustering links divergent viruses (cf. S7 Fig, panel C), implying similar feature usage even among viruses considered unrelated in the taxonomy. Numerical data underlying this figure can be found at https://github.com/nardus/zoonotic_rank/tree/main/ [file pbio.3001390.s010.pdf]

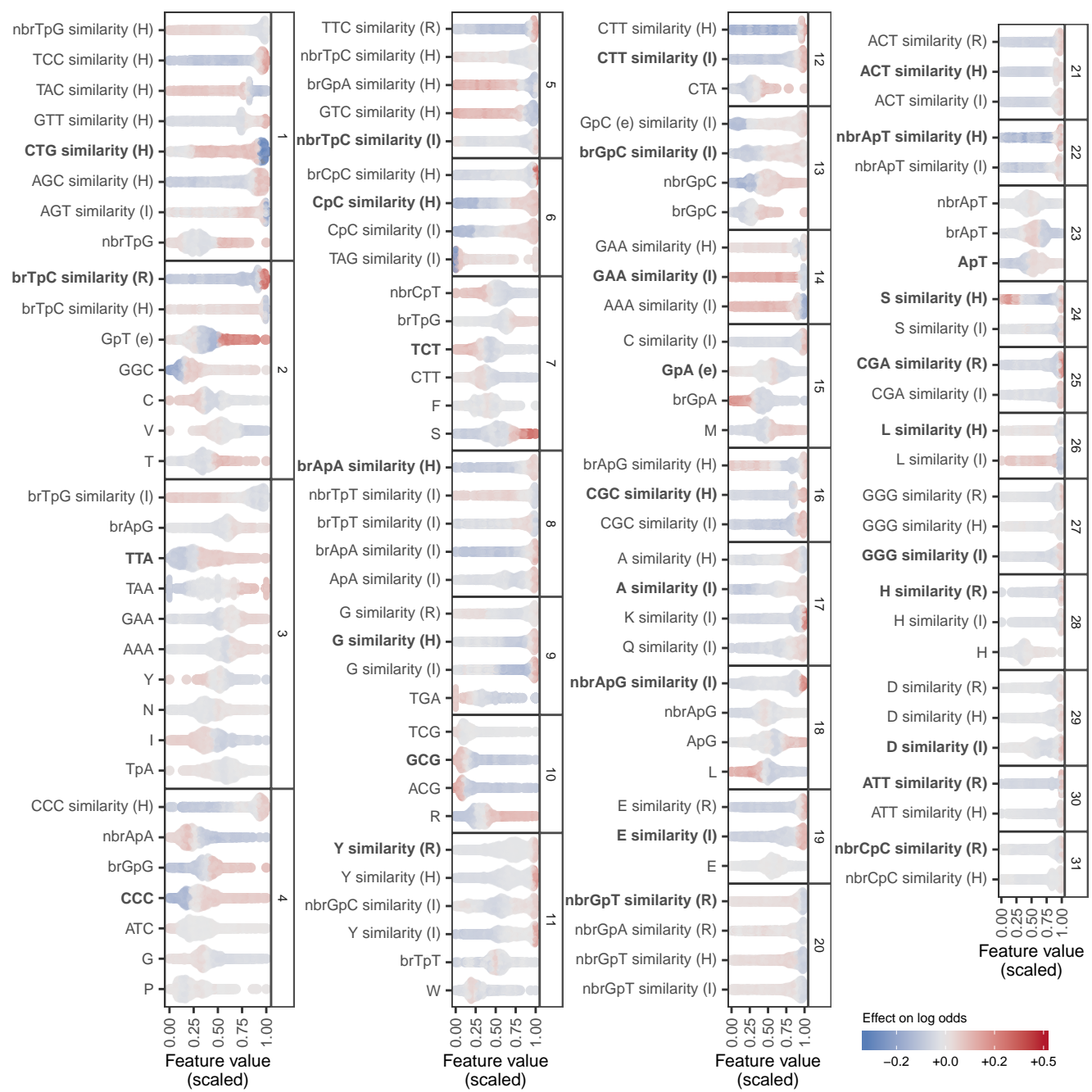

Supplement: S10 Fig — Each panel indicates a discrete cluster of correlated features, numbered by relative importance (see Fig 2D). Points are colored to show the effect of each feature on the predicted log odds that an individual virus infects humans (i.e., the SHAP value for that feature for a given virus, derived from the combined genome feature–based model, and averaged across all 1,000 iterations). The x-axis shows observed feature values (scaled to lie between 0 and 1 to place all features on the same scale), with points jittered to approximate local density where they overlap. Feature abbreviations follow a similar pattern to those in S9 Fig, except that bridge and nonbridge dinucleotide biases are preceded by “br” or “nbr,” respectively. Capital letters in parentheses indicate the set of human genes used as baseline to calculate measures of compositional similarity (I = ISG, H = housekeeping genes, and R = remaining genes). Names ending in “(e)” represent features calculated across the entire genome; all other features were calculated with reference to coding sequences only. The exemplar of each cluster is highlighted in bold. Numerical data underlying this figure can be found at https://github.com/nardus/zoonotic_rank/tree/main/FigureData (doi: 10.5281/zenodo.4271479). SHAP, SHapley Additive exPlanations. (PDF) [file pbio.3001390.s012.pdf]

Zoonotic potential: Low Medium High Very high

**A**

Sampled host order

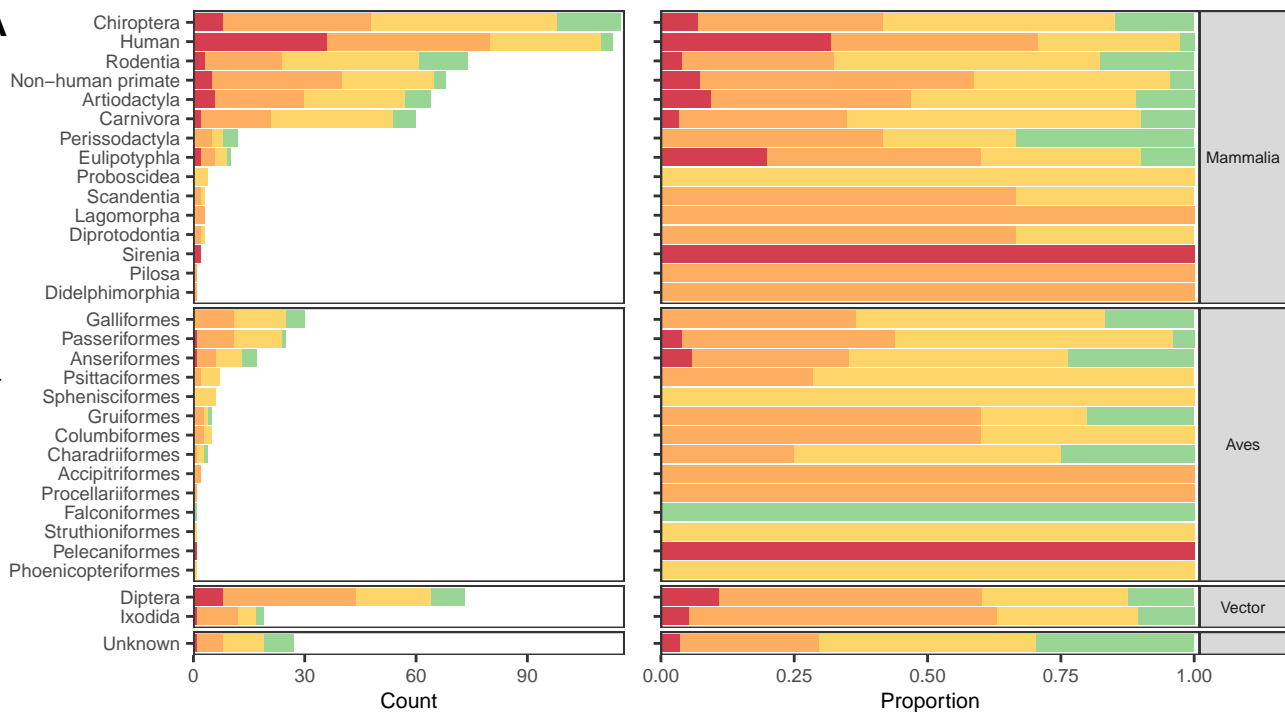

**B**

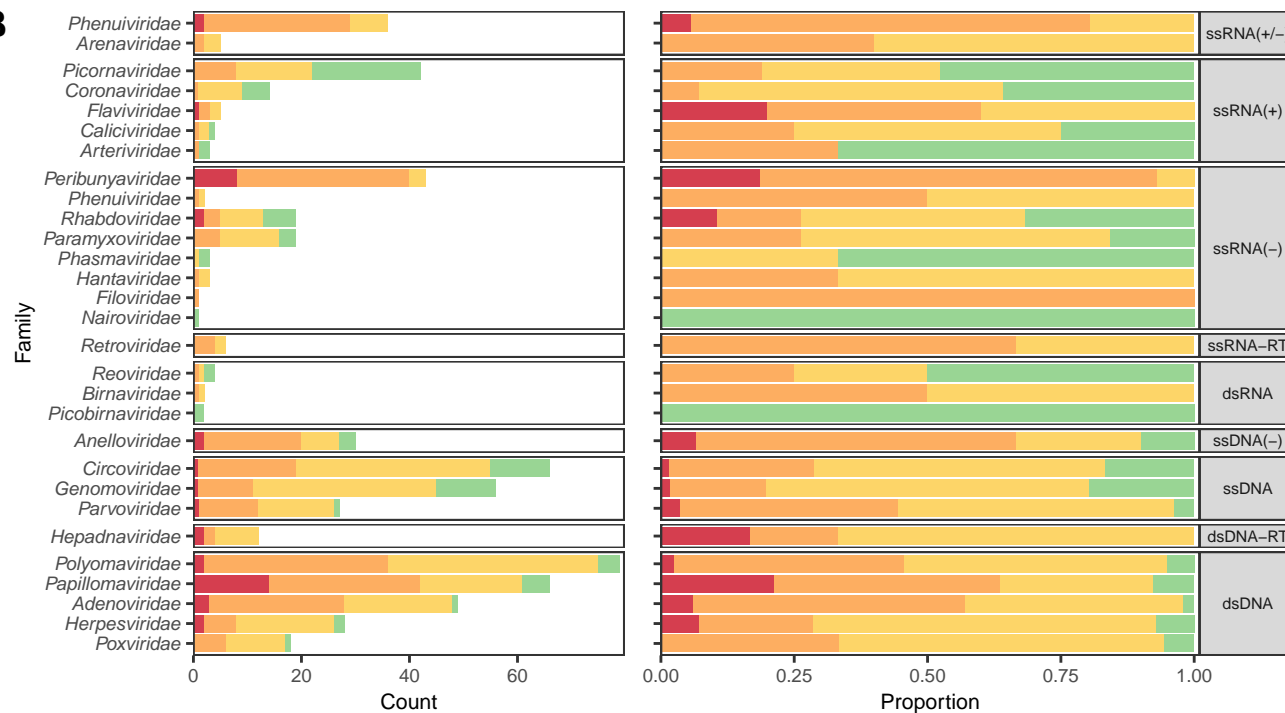

Supplement: S11 Fig — (A) Distribution of zoonotic potential category assignments by sampled host, for all viruses in Fig 3A. (B) Distribution of zoonotic potential categories among viruses from Fig 3A that were sampled from nonhuman hosts, arranged by viral family and genome type. Numerical data underlying this figure can be found at https://github.com/nardus/zoonotic_rank/tree/main/FigureData (doi: 10.5281/zenodo.4271479). (PDF) [file pbio.3001390.s013.pdf]

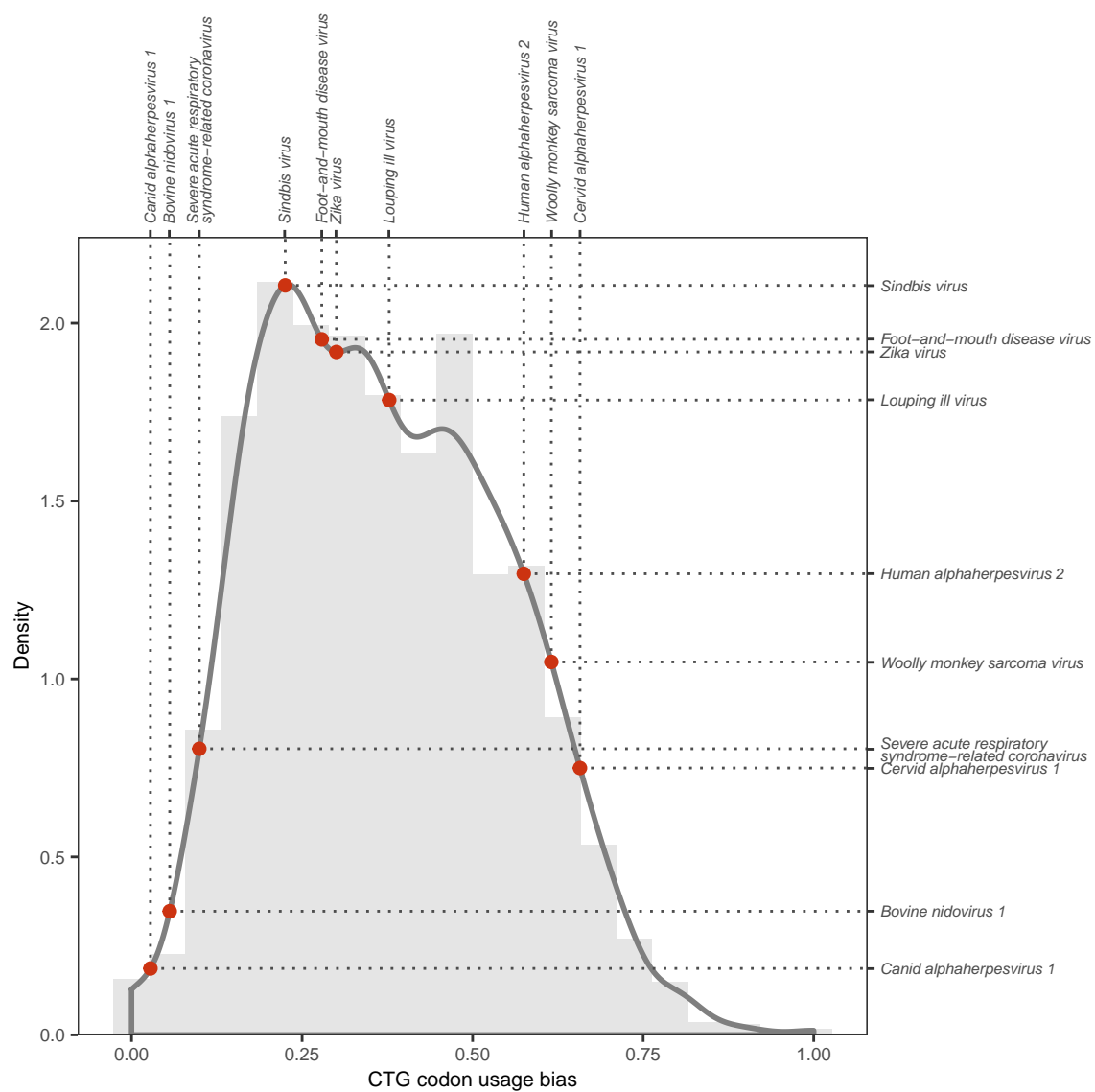

Supplement: S12 Fig — A histogram (light gray bars) shows the distribution of CTG codon usage bias observed among human non-ISG housekeeping genes. This distribution was used to estimate an empirical probability density function (dark gray line). Evaluating this function for the values of the same feature observed for specific viruses gave the final similarity scores (red dots, with the x-axis representing observed values and the y-axis the new similarity score). Using this similarity score as representing an estimate of how well each virus genome mimics the particular population of human genes resulted in rearrangement of viruses that may help to predict the ability to infect humans. Because similarity scores were calculated via a density function, viruses with feature values significantly outside the range observed for a given set of human genes received a score of 0, but such cases were rare (0.025% of calculated similarity scores, affecting 5% of similarity-related features). Numerical data underlying this figure can be found at https://github.com/nardus/zoonotic_rank/tree/main/FigureData (doi: 10.5281/zenodo.4271479). (PDF) [file pbio.3001390.s014.pdf]

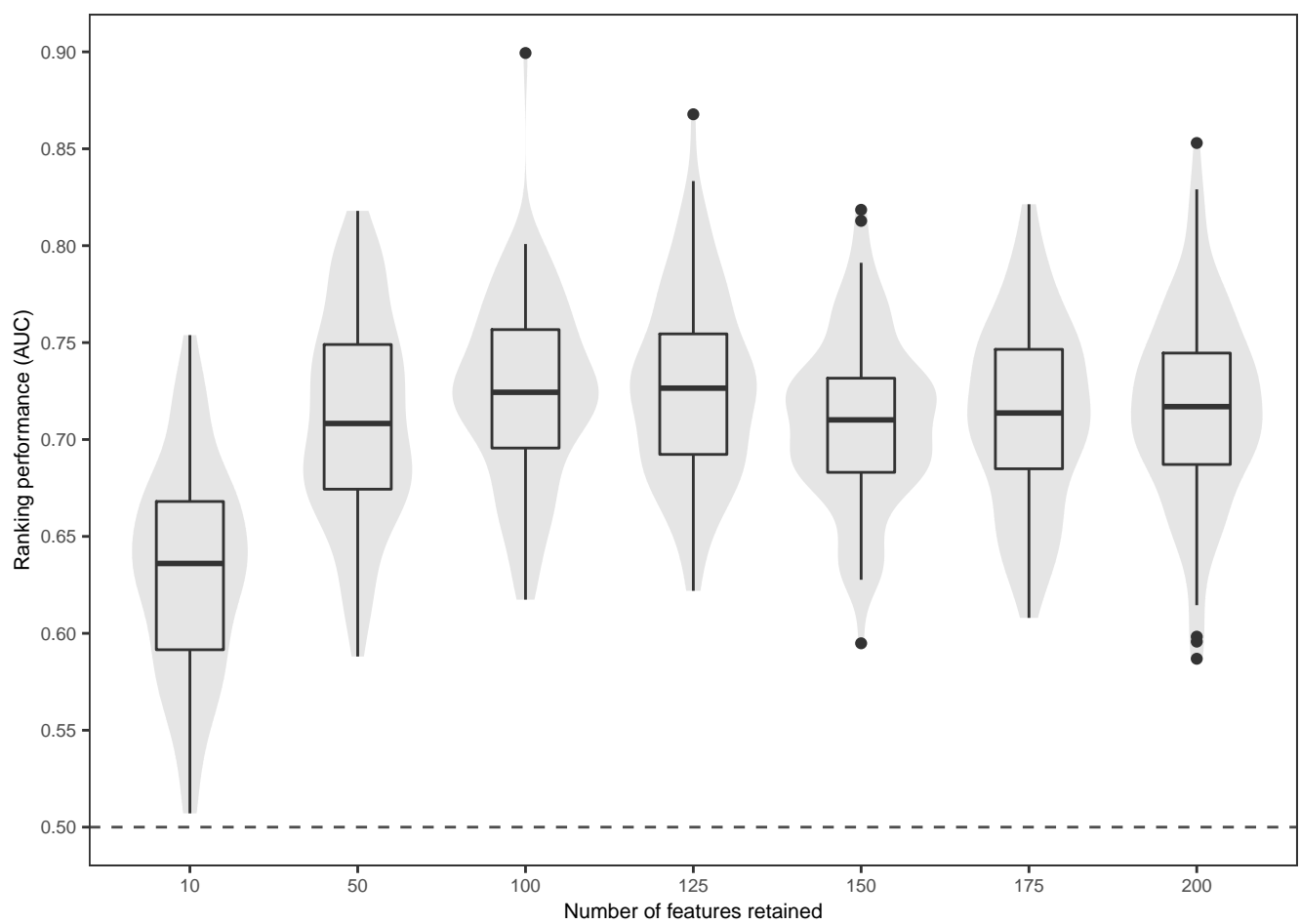

Supplement: S13 Fig — Boxplots and shaded areas illustrate the distribution of AUC values obtained when training classifiers on 100 random test:train:calibrate splits. For each set of classifiers, the top N most predictive features selected from among all feature sets were retained, with N indicated on the x-axis. Numerical data underlying this figure can be found at https://github.com/nardus/zoonotic_rank/tree/main/FigureData (doi: 10.5281/zenodo.4271479). AUC, area under the receiver operating characteristic curve. (PDF) [file pbio.3001390.s015.pdf]
