# Supplementary material for: Identifying and prioritizing potential human-infecting viruses from their genome sequences
Source: PLoS Biol. 2021 Sep 28;19(9):e3001390. doi: 10.1371/journal.pbio.3001390 (PMC8478193; doi:10.1371/journal.pbio.3001390)
Supplement: S9 Fig — Discrete clusters of features were obtained using affinity propagation clustering based on the Spearman correlation between all features present in the final model. Clusters are numbered to match their relative importance as defined in Fig 2D. Distances between features are illustrated in 2 dimensions, obtained by multidimensional scaling of the pairwise correlation matrix. Individual clusters are shown to different scales in (A) for readability, while (B) shows all clusters on the same scale. All points are shown connected to the exemplar feature of that cluster, which is also indicated in bold font. Colors indicate the magnitude of each feature’s effect on the combined genome feature–based model’s output, calculated as the mean of absolute SHAP values across all viruses in the training data, and averaged across all 1,000 model training iterations. Feature names abbreviated to a single letter indicate amino acid biases, while 3-letter codes written in capital letters indicate codon biases. Dinucleotide biases are abbreviated in the form “CpG” and were calculated separately for codon bridge positions (abbreviation preceded by “b,” e.g., “bCpG”), nonbridge positions (preceded by “n,” e.g., “nCpG”), and also across all coding sequences of a given genome (no prefix, e.g., “CpG”) or across the entire virus genome (suffix “.e,” e.g., “CpG.e”). Numerical data underlying this figure can be found at https://github.com/nardus/zoonotic_rank/tree/main/FigureData (doi: 10.5281/zenodo.4271479). SHAP, SHapley Additive exPlanations. (PDF) [file pbio.3001390.s011.pdf]

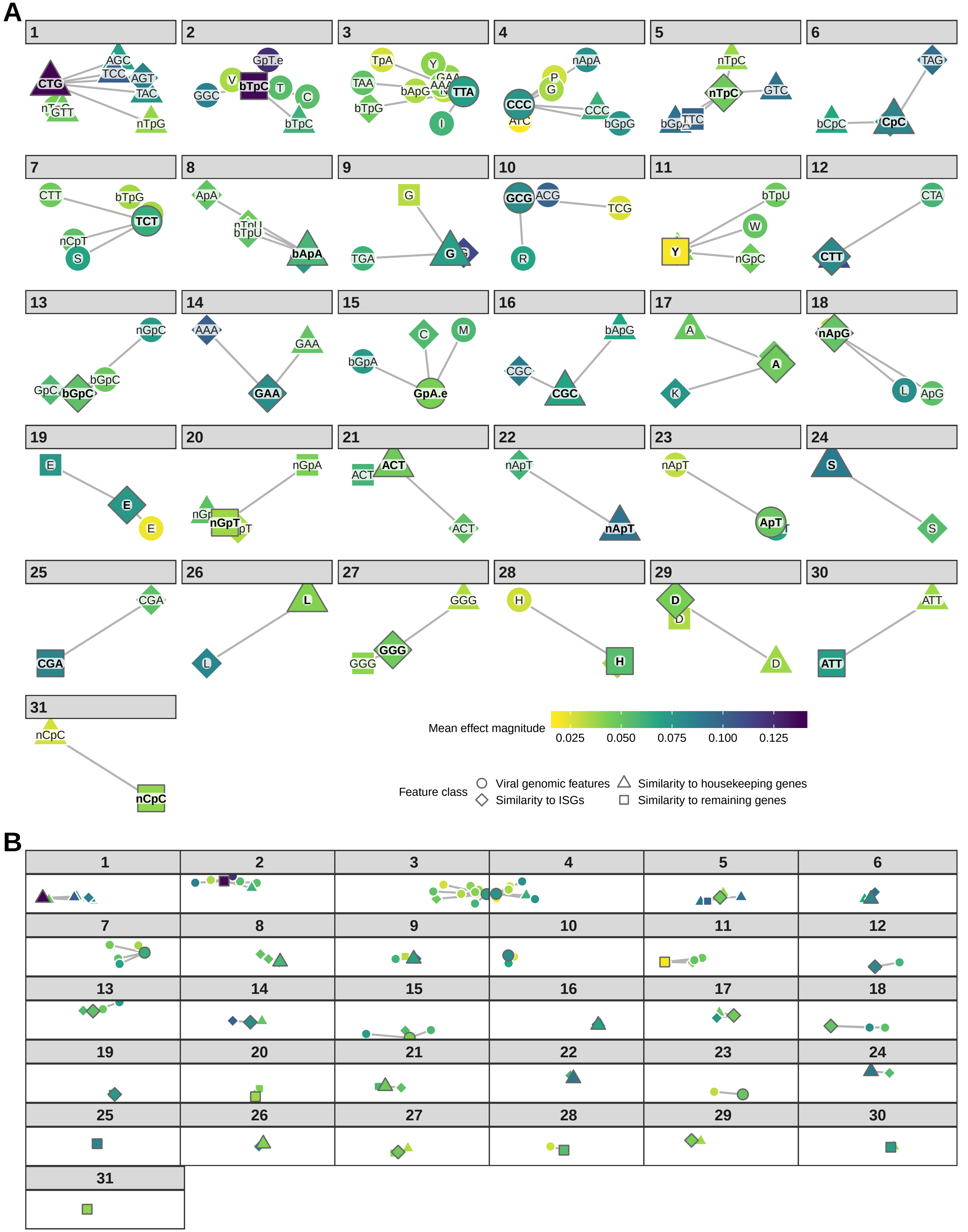
A
1
CTG
AGC
TCC
AGT
TAC
GTT
nTpG
2
GpT.e
V
bTpC
GGC
T
C
bTpC
3
TpA
Y
TAA
bApG
TTA
bTpG
I
4
nApA
P
G
CCC
CCC
bGpG
5
nTpC
nTpC
GTC
TTC
6
TAG
bCpC
CpC
7
CTT
bTpG
TCT
nCpT
S
8
ApA
bTpU
bApA
9
G
TGA
G
10
GCG
ACG
TCG
R
11
bTpU
W
Y
nGpC
12
CTA
CTT
13
nGpC
bGpC
GpC
bGpC
14
AAA
GAA
GAA
15
C
M
bGpA
GpA.e
16
bApG
CGC
CGC
17
A
A
K
18
nApG
L
ApG
19
E
E
E
20
nGpA
nGpT
21
ACT
ACT
ACT
22
nApT
nApT
23
nApT
ApT
24
S
S
25
CGA
CGA
26
L
L
27
GGG
GGG
GGG
28
H
H
29
D
D
D
30
ATT
ATT
31
nCpC
nCpC
Mean effect magnitude
0.025
0.050
0.075
0.100
0.125
Feature class
Viral genomic features
Similarity to ISGs
Similarity to housekeeping genes
Similarity to remaining genes
B
1 2 3 4 5 6
7 8 9 10 11 12
13 14 15 16 17 18
19 20 21 22 23 24
25 26 27 28 29 30
31
