# Supplementary material for: Identifying and prioritizing potential human-infecting viruses from their genome sequences
Source: PLoS Biol. 2021 Sep 28;19(9):e3001390. doi: 10.1371/journal.pbio.3001390 (PMC8478193; doi:10.1371/journal.pbio.3001390)
Supplement: Attachment — Submitted filename: Zoonotic_rank_response_to_reviewers.V5.docx [file pbio.3001390.s016.docx]

# Point-by-point response

Note that line numbers below refer to main text document *without* changes tracked.

## Reviewer #1:

In "Identifying and prioritizing potential human-infecting viruses from their genome sequences" Mollentze et al. use machine learning approaches to examine the potential for using various genomic features of viral genomes to predict whether the virus will be able to infect humans. Although I cannot evaluate the details of the machine learning approach used, the manuscript is well written and overall, the analysis appears sound.

The main contribution of this study is demonstrating that there is relevant information within viral genomes for predicting the potential of a virus to infect humans, and that this includes additional information beyond that provided by broad-level taxonomy and phylogenetic similarity. There are two primary, potential implications of this: 1) perhaps viral genomes could be used to prioritize new viruses with zoonotic potential and 2) the genome features that are informative about the potential to infect humans could help us to better understand the adaptation of viruses to certain host species.

The first of these is the primary focus of this manuscript. However, in practice, I question just how useful this model will be for this type of prioritization given that 25% of the 'negative' training set ("No known human infections") is still predicted to be of high or very high priority. Similarly, >25% of human infecting viruses are categorized as low or medium priority. Given the high number of new viruses that are regularly being identified, and assuming that most will not be capable of human infection, it's not clear to me that using the output of this model will significantly improve our ability to prioritize high risk viruses. The results certainly don't seem compatible with the implications of this statement: "The performance of our models, while imperfect, means that many potential zoonoses can be identified immediately after virus discovery and genome sequencing." Say 10% of new viruses characterized from animal hosts have the potential to infect humans (probably an overestimate), then only about ~23% of the viruses selected by the model to be high or very high priority would actually be capable of infecting humans. Sure, the model will identify some, but how can users distinguish between the true and false positives?

We have made substantial additions to the text (including several new figures) which aim to make the application of our models for guiding decision-making for research and surveillance more concrete.

As a first point, we think it is relevant to view the performance of our model in the context of the current state of the art in the field. Although the performance of our model is imperfect, it represents a considerable quantitative and qualitative improvement over current approaches to triaging newly discovered viruses. At present, the only available options for choosing which novel viruses to study further are either doing so randomly, using phylogenetic information, or using a combination of host traits, interpolated data, and expert opinion. Random screening would have an AUC of 0.5. As we show in figure 1, using taxonomic or phylogenetic information is only marginally better than this, and can actually be worse than random guessing depending on which viruses are already known (as indicated by the AUC of models trained on some training datasets being below 0.5). That last approach, recently published in PNAS (Grange et al. 2021, doi: 10.1073/pnas.2002324118), provided no metric of predictive performance. However, we argue this approach has reduced utility since it relies on many variables which, being unknown at the time of virus discovery, must be borrowed from related viruses; therefore incorporating the phylogenetic information which we show here is uninformative and potentially misleading. In this context, the AUC of 0.773 seen in our manuscript is a large improvement.

We appreciate the reviewer’s concern about false positives and false negatives, but think the provided metrics of sensitivity and specificity make a strong case for the utility of our models. For example, if only viruses in the very high zoonotic potential category can be characterised further, then our results show that a clear majority (at least 77% of the viruses screened) will indeed turn out to be zoonotic. Similarly, if all viruses in the very high and high zoonotic potential categories were screened (41.9% of all viruses), then we would expect 70.5% of the zoonotic viruses to have been found, despite zoonoses comprising the minority of newly discovered viruses (this is now explained on lines 178 – 189). This is the reasoning behind our statement on “many potential zoonoses”, which in fact constitutes the majority of zoonoses. A further strength of our approach is that continuous rankings of zoonotic potential allows researchers to inexpensively characterise as many viruses as desirable from sequence data and work down the priority list, knowing that the set of viruses characterised first will be enriched for human-infecting viruses (now illustrated in figure 1D). As now illustrated in figure S6, alternative prioritisation approaches available immediately after virus discovery require considerably more effort to identify an equivalent number of human-infecting viruses (e.g., a 1.7-fold increase in effort to identify the first 50% of human-infecting viruses).

The reviewers’ point about false positives is fair, but since our approach would reduce false positives considerably better than any approach available, it has a clear benefit over current practice. Finally, as discussed on lines 103 – 107, at least some of the high-ranking viruses which currently appear to be false positives may in fact be capable of infecting humans, making the reported specificity values conservative. Indeed, of the 14 viruses not currently known to infect humans which were placed in the very high priority category, three already have serological evidence of infecting humans (line 174 – 178).

We now also illustrate these points better by using a much larger case study in figure 3, in which 645 animal-associated viruses that were excluded from training were reduced to just 41 very high risk viruses. We have also added a comparison to the best available current model for which performance statistics could be calculated (Olival et al. 2017, Nature, doi: 10.1038/nature22975), showing that our models perform considerably better using the data that is likely to be available for newly discovered viruses and perform at least as well as this existing approach for well-studied viruses with more data available (lines 402 – 411). We have also now taken care to avoid implying a direct application of our results to policy, emphasising that our rankings are intended to narrow the number of viruses requiring further, complementary research on zoonotic potential, such as cell culture infection tests or serological screening of high-risk human populations (lines 423 - 437, but also lines 52 – 55, 179 – 189).

I think it would have been more interesting to see a deeper exploration of the genomic features shown to be associated with human infecting viruses.

A comprehensive understanding of genomic features is unfortunately very difficult to obtain at present, since these features remain poorly understood and many of the features are correlated with each other. This might be due to co-evolution or trade-offs between features, or because the currently-used measures of composition are relatively crude. For example, the best-studied dinucleotide, CpG, is known to be form part of an unknown motif recognized by ZAP. As such, CpG frequency gives some indication of which RNA molecules will be recognized by ZAP, but it is a relatively crude measure since not all CpGs will be in the correct sequence context to be targeted.

We therefore believe it would be premature to speculate on mechanisms at this stage. However, to accommodate the reviewer’s request, we expanded the supplementary text (S1 Text) and also the main text (line 235 – 253), exploring general patterns among features and emphasizing the value of further research on the features we identified, ideally using targeted experiments to avoid the correlation between features we identify in observational data. We also added a paragraph to the discussion emphasizing the significance of our finding that some features appeared to predispose human infectivity across many divergent viruses, which we agree did not receive enough attention in the original submission. We now explain the significance of this discovery for the generalizability of our models and for understanding virus host-adaptation more broadly (line 438 – 455). The phenotypic effects of viral genome composition have previously been of interest primarily in the context of virus attenuation in vaccine research. As the reviewer points out, our results highlight that deeper understanding of viral genome composition also has broader implications for understanding host range.

### Other concerns:

1. The authors clearly describe how they determine which viruses they consider to be able to infect humans, but they don't clearly describe where they obtained the list of 861 viral species used or how human infecting viruses were categorized as primarily human viruses or zoonotic. These aspects of the methods need to be clarified.

The final list of 861 viral species was obtained by merging datasets from three published studies, with the final dataset including all species for which a previous attempt at finding evidence of human infection has been made. We have improved the explanation of how this list was generated on line 489 – 496.

The distinction between primarily human-transmitted and zoonotic viruses was based on a previously collected dataset of virus reservoirs. This is now explained on line 497 – 502.

2. For the novel viruses, it is important to demonstrate that these are really new species (and genomes) and not simply re-categorization of existing/known strains.

We apologize for the confusion that our wording introduced here. The case study now includes all relevant viruses currently recognized as species but not present in our training data (which is the key point, rather than when species were discovered). To ensure that name changes or re-categorizations did not affect our results, we matched all names of viruses in our training data to the latest ICTV taxonomy, allowing us to exclude these from the case study (now explained on line 685 – 687).

3. It is specified that for the novel viruses tested, viruses were only included id they were "from families known to contain species that infect animals." Was this same criterion also used for the 861 species included in the training/testing set? If not, it seems that this is a biased subset to use for such testing.

Yes, the training data was also restricted to animal-infecting virus families. We have expanded on this on lines 684 – 685.

We further note that this restriction was needed to avoid biasing performance metrics upwards. Based on earlier results showing major compositional differences between e.g. vertebrate- and invertebrate-associated viruses (Babayan et al. 2018, Science, doi: 10.1126/science.aap9072), virus families associated exclusively with hosts that are very distant to humans (e.g. plant viruses or bacteriophages) would be easy to identify as not human-infecting by genome composition-based models. However, good performance on such viruses is misleading, since we would not expect end users to have much doubt about whether viruses from such families can infect humans. We did however include invertebrate- and plant-associated viruses when they occur in a family which also contains animal-infecting viruses (e.g. *Rhabdoviridae*) as metagenomic detection of new species in these families may be more difficult to separate into animal and environmental viruses *a priori*, and we therefore needed to train the model to not produce incorrect results when applied to such viruses.

4. ~28% of the novel genomes (71/256) belong to Anelloviridae (Torque Teno viruses), and as noted in the manuscript, no viruses from this group were including in the training/testing set. This seems strange to me. These species certainly existed prior to the latest ICTV release.

This family was indeed known before, but was absent from all three of the published datasets merged to form our training data. As described above, we have now replaced the “novel virus” case study with one which includes all currently-recognized species which were missing from our training data. This set was restricted only by (a) keeping to the 38 families of viruses known to contain animal-infecting viruses, and (b) retaining only viruses sampled from birds, mammals, or potential vector orders or for which the host sampled was not reported. This last restriction is needed to make the host information added to figure 3 readable.

5. One confusing aspect of the decision to only include one representative virus from each ICTV species is that certain species are composed several different strains, only some of which have been associated with human infections. For example, Betacoronavirus 1 includes the human endemic virus HCoV-OC43 and also several strains only associated with infections of other mammals. Why was this not taken into account when choosing the representative genome for each species? I would also like to see some discussion of the potential impact of this on model predications.

Our data sources only provide information on zoonotic status at the species level (line 100 – 103). While it may be possible to resolve human infection-ability to the strain level for a small number of well-studied virus species, this is not generally the case. Unless the virus has been subject to intensive surveillance, we would not be able to distinguish strains which truly cannot infect humans from ones which simply have not been observed to do so due to either random chance, lack of ecological opportunity, or a lack of testing. Further, as discussed in the manuscript (lines 57 – 61), we would not have been able to evaluate the expected performance of models on entirely novel species fairly had the training data included multiple representatives of each species and we believe this approach would have hampered the ability of our model to generalize across virus groups.

For human-infecting viruses, we selected the genome of the variant linked to humans whenever multiple distinct reservoirs were known. For all other viruses, we used the NCBI reference genome, which seems like the most neutral choice. As requested, we have added a discussion of the implications of the species-level nature of our data (lines 456 – 468), highlighting that the ranks produced here apply only to the specific genomes tested. We now also include the accession numbers evaluated in our table of virus ranks (S1 Table).

6. Of the 43 novel viruses predicted to be unlikely to infect humans, have any been shown to cause human infections?

We now include information on human infection in figure 3 and highlight 3 viruses from the new case study which are known to infect humans but which were mis-classified as low zoonotic potential on line 263 - 266. Related to this query, we carried out a new statistical analysis of the holdout data (i.e., the case study in figure 3) which confirmed that viruses which had been sequenced from human samples were more likely to be predicted to be zoonotic by our models. This provides a systematic test of our model’s ability to distinguish among viruses not available during training (new Figure 4), providing further external validation of our model’s performance.

7. 14/19 of the unknown viruses predicted to be in the very high priority were Torque Teno viruses (anelloviruses). In total, 71 of these viruses were included in the novel set. What proportion of these 71 were isolated from humans? What is the distribution of known human-infecting anelloviruses in the various prediction categories.

The proportion of viruses classified into each priority category is now shown summarised at the family level in figure S11.

8. The conclusion that SARS2 ranked "considerably higher" than SARS1 in the analysis show in Fig 3C seems potentially a bit misleading. The distributions shown are largely overlapping. Is there a significant difference between these distributions? Given the relatively subtle difference between SARS1 and 2, and the fact that several other coronaviruses, not known to cause human infections, are also ranked similarly to SARS2 in Fig 3C, I think it is important that this statement in the abstract is tempered: "…could have identified the exceptional risk of SARS-CoV-2 prior to the emergence of the first SARS-related coronavirus in humans."

We agree. In the main text, we have toned down the relevant text to simply state that SARS-CoV-2 and very close relatives were placed in a higher priority category from the majority of sarbecoviruses tested, while pointing out that confidence intervals overlap (lines 370 – 374). We have also modified the abstract to avoid implying a major difference between SARS-CoV and SARS-CoV-2. However, the key result referred to in the abstract is that our models could have detected SARS-CoV-2 as a risk even without knowledge of earlier SARS-CoV-related outbreaks. This is an important signal of the generalizability of our models, which arises from their use of signals which appear to influence human infectivity across divergent viruses. We have expanded our explanation of this result on lines 374 – 381, highlighting that only models trained while excluding all SARS-CoV-related genomes were used to produce the reported SARS-CoV-2 predictions. The ability of our model to find high risk zoonoses even when close relatives have not been identified as zoonotic is crucial and sets our approach apart from all other strategies for viral risk assessment.

9. For Fig. 3A, please provide some indication of from which host species each of the viruses was isolated.

Thank you for the suggestion. This has now been added, and we have also added a new figure 4 and text on lines 294 – 315 highlighting that – with the exception of viruses from humans and other primates – the host or vectors sampled had no detectable influence or predicted risk. We believe this new result strengthens our manuscript by contributing a new line of evidence addressing the controversy regarding the proclivity of certain animal orders to host zoonotic viruses. As discussed on line 305 – 308, the effects on human and primate origin provide a further line of evidence for the utility and biological basis for our models.

10. For Fig 3C, Need to provide meaning of circles and error bars in legend.

Done (line 289 – 292).

11. Fig. S3. The meaning of the dark grey is not clear. Please describe.

This is now explained in the caption of the figure (now figure S4, caption on line 906 – 917).

12. The analysis shown in Fig. S9 should include the coronaviruses from animals that are most closely related to SARS-CoV-2. For example: RaTG13, CoVZXC21, CoVZC45 and the pangolin-isolated viruses from 2017 and 2019.

We have added RaTG13 and the pangolin-associated viruses to this figure (now figure 5B). We were unable to include CoVZXC21 and CoVZC45, since our phylogeny is based on the most conservative alignment from Boni *et al.* 2020 (Nature Microbiology, doi: 10.1038/s41564-020-0771-4), where these recombinant sequences were excluded to maximize alignment length while ensuring the alignment was free of incongruent phylogenetic signals. However, we believe that figure 5 now gives a good overview of the distribution of scores which can be expected from currently known sarbecoviruses, as all viruses in the clade which includes SARS-CoV-2 score similarly and slightly higher than all other sarbecoviruses.

## Reviewer #2:

I really liked this paper, and I think it could potentially be published in PLoS Biology. The introduction and results read really well. The discussion needs more context of what's been done in the past. The methods are all AI, and this needs a lot more explanation as many readers of PLoS Biology, all emerging disease ecologists, and even most bioinformaticians will not be familiar with these approaches. It's not enough to cite a paper in the methods and say that some algorithmic/statistical approach was taken. The reader needs to know exactly what this approach does so they can put it in context of the paper's biological assumptions and conclusions.

I don't work in AI or machine learning. Please keep this in mind as you read the comments below as some may sound elementary. However, the paper does need to reach a biology audience with no background in AI/ML.

### Major points

1. Most important comment to address is how the probability p was calculated (the probability shown in Figure 3, and maybe in Figure 2A). I tried to follow this and didn't have time to go to the source material. Is p the mean across the best-performing 10% of models as in the Fig 3 caption? If this is the right definition, then if you want to categorize a new virus, which model do you choose from these best 10%? Is p the output probablity described on line 334? Line 372 suggests that p comes from a logistic regression approach, but maybe this is a different p.

The probability p is the output probability averaged across the best 10% of model iterations. This set of models/iterations are used for both known and holdout viruses, giving an indication of the variability or uncertainty in predictions stemming from the fact that the currently known viruses used to train the models represent only a very small fraction of the virus diversity likely to exist. Thus, for novel viruses one would not choose a specific model, but instead rely on the range of predicted probabilities from models whose performance has already been evaluated on the test data, as we do for the case studies in figures 3 and 5. We have expanded the explanation in the caption of figure 3 (line 289 – 292), and also now mention the use of this mean probability much earlier in the text (at the first mention of the bagged model, lines 154 – 157). We also corrected the y-axis label of figure 2A to be consistent with figure 3. Finally, the relevant section of the methods (line 599 – 612) has been rewritten to improve clarity on this point.

2. line 111: what are these 100 or 1000 models? Are these different parameterizations of the same statistical learning algorithm, each parameterization corresponding to a different training set? Is it common in machine-learning papers to label these as different "models"? Line 312 says a "range of models" but this seems to refer to the nine "models" or "feature set combinations" in Figure 1A. Is this correct?

Thank you for pointing out this inconsistency. We have now taken care to use the word “model” only for classifiers trained on different feature sets, using “iterations” to refer to the replicated trained models (i.e., the same model trained on 1000 resampled training datasets to measure uncertainty).

3. How many features are there? Figure 1 shows very clearly how good the prediction is when certain feature groups are included or excluded. But, how many features are in the "Similarity to ISGs" feature set, for example? If a virus is 10kb long, do we choose some length maximum, or some number of windows to look at? And then do we take these fragments and find best local alignments to all N=2054 ISGs? Do we take the score from this local alignment as a similarity score and input it into the ML algorithm? Please just tell me using a back-of-the-envelope calculation (if possible) how many ISG features there are for a 13KB influenza A genome. Later in the paper you settle on N=125 features to use, and I had no idea whether the total number of starting features was nine (Figure 1A), one thousand, or one million.

The number of features calculated is constant regardless of genome size, because all features used here represent summary statistics of genome composition. We now consistently use the term “feature set” when referring a collection of related features (e.g., the set of features measuring similarity to ISGs), and report the total number of features in each such feature set (line 520, 524, 546, and 558).

4. What is a SHAP value? In simple language, made understandable to a non-AI moderately quantitative scientist.

We have expanded the explanation on line 193 – 199 to explain that SHAP values are a way of measuring the relative influence of each genomic feature on the model prediction produced for a given virus. These values therefore give insights into why certain predictions were made, and hence allow us to explore the patterns identified by the model. While this is somewhat indirect, this approach is needed because the machine learning models we used prioritise predictive performance over statistical inference. Improving the interpretability of and comparability between machine learning models is an active area of research, and SHAP values are the cutting edge.

5. What is a "per-virus effect size" of a feature? Is this the effect size in Figure 2? If not, please define both of these.

This explanation has been improved, removing this term (see previous comment).

6. Page 6: 70% of viruses categorized correctly. Is this good? Should we be aiming for something like 90% or 95%? Is this normal for AI methods on complex problems like this one? Is it better than what other approaches have achieved? I feel like this point needs some serious discussion. You can't isolate a novel rare virus in the middle of a rain forest and tell the local animal health department that you're 70% sure it would infect humans. If all approaches in the past have yielded 70% predictability, then maybe the current paper is not much of an advance.

As discussed in response to a related query by reviewer 1 above, we have added text and two new figures to better explain the utility our models (lines 178 – 189, figure 1D, and figure S6). While the performance of this model is imperfect, it can rank-order viruses in a meaningful way using ‘real’ data (i.e., without relying on filling gaps using data from related viruses, which may be phenotypically distinct, or expert opinion, which may be biased; both of which are utilized by existing models). This means that nearly 70% of human-infecting viruses will be found within the first 40% of viruses in our ranked list. Thus, subsequent confirmatory tests screening viruses in the order suggested by our models would find the majority of human-infecting viruses more efficiently than is currently possible (figure 1D). We have now taken care to emphasise in several places throughout the manuscript that these models are not a standalone solution. These models are not able to identify all human-infecting viruses, but since a virus genome will have to be generated for all other virus characterisation steps in any case, obtaining a genome-based prediction comes at no additional cost, and allows a better allocation of resources in cases where not all of the viruses identified can be characterised in detail. We address the performance of our approach compared to others in our response to the next comment.

7. In general, how much of an advance is this over previous work done in this area. I see that some of this previous work is described in the introduction, but more detail (either in intro or discussion) would be helpful for the reader to understand the need for a better approach. At some point during the 2007-2012 period, many scientific groups were receiving funding for viral discovery, viral chatter, identification of potential pandemic viruses, identification of zoonotic viruses (see Mark Woolhouse, Nathan Wolfe, Peter Daszak, Paul Kellam, Peter Simmonds). Did this work go anywhere? Did all the deep sequencing approaches work for either virus discovery or virus characterization? What were the biggest drawbacks of the goals set out during this period? Were all the nice-looking diagrams in Nature and Science showing different risk levels of zoonotic jumps correctly put together? Did any of these groups have a 70% accurate prediction method for novel viruses? I am not one of the five authors listed above, and I have no interest or stake in the success/failure of these past projects. This is a genuine question.

The virus discovery work mentioned is ongoing, and e.g. the PREDICT consortium have reported more than 700 putatively new virus species so far. Despite all this discovery work however, the question of what to do with the novel viruses being discovered remains unanswered. While preparing this revision, an approach using expert opinion-based risk factors to prioritise novel viruses was published by the PREDICT consortium (Grange et al. 2021, PNAS, doi: 10.1073/pnas.2002324118). However, this approach appears to require extensive knowledge about the ecology and virology of viruses before a reliable assessment can be made, making it unsuitable for application immediately after virus discovery. Unfortunately, Grange et al. include no formal validation or measurements of accuracy, making it impossible to compare their approach to our models.

Beyond Grange et al., there has been very little previous work in this space – general models focussed on identifying risk factors associated with zoonotic ability have been published, but these have not been evaluated for prediction accuracy. To allow comparison, we have now evaluated the most comprehensive of these models (from Olival et al. 2017, Nature, doi: 10.1038/nature22975) using a cross-validation approach similar to that used for our own models. This model performs similarly to our best model (AUC = 0.770; our best AUC = 0.773) on viruses with known human infection status but requires considerably more information (e.g., the range of hosts that each virus infects and publication counts) making it unsuitable for newly discovered viruses. We further show that if this earlier model were applied using only the information that could conceivably be available or reliably inferred at the time of virus discovery, it performs considerably worse (AUC = 0.647) than our approach, which by comparison requires only a genome sequence (AUC = 0.773, as above). The fact that our approach is equally or more accurate, more generalizable, and works from just a single genome rather than variables which require years of research to collect is a major advantage. This is now discussed on lines 402 – 422.

In the machine learning literature, we are aware of 1 publication and 1 preprint claiming to identify zoonotic viruses from metagenomic sequence data, but as mentioned on line 56 – 61, these models were trained and evaluated using large numbers of representatives of the same virus species. This makes it impossible for us to evaluate their potential accuracy on novel virus species, since the statistics they report were generated by models which had seen other examples of the very species they are being tested on. However, as these models have very different aims and were not designed for use on novel species, it would be unfair to criticise them for these design choices in the discussion of the present manuscript.

### Minor points

8. Abstract - "prior to the emergence of" .. SARS1 or SARS2? Maybe it's simpler to say here that your approach "is able to correctly classify SARS-CoV-2 as having zoonotic potential based on genomic data alone"

This sentence has been modified for clarity. Our key point here is that our models can anticipate this risk even when they are trained on data which does not include any earlier SARS-CoV-related genomes, which is key to their generalizability and utility. This result is also now explained more extensively on lines 374 – 381.

9. Abstract - "developed machine learning algorithms" .. did the authors develop new algorithms, adapt existing ones, use standard algorithms inside R-packages?

We agree. Most of the novelty in our approach is in the extraction of features from genomes which then are fed into standard machine learning algorithms. This has been changed to “developed machine learning **models**” (line 24).

10. Introduction, second para, "Empirical and theoretical lines of evidence suggest such signals might exist [8,9]." - just a couple more sentences of detail here please on what these lines of evidence are.

This has been added on lines 62 – 70.

11. line 105: tested independently? Or tested seperately?

This has been changed to “separately” (line 147).

12. line 106: "Combining all genome composition features.." -- I would write "combining both types of genome composition features.."

Agreed, this has been changed to “Combining all four genome composition feature sets…” (now line 150).

13. what is the definition of the "priority categories" (lines 115-116)

The definition of priority categories have been moved from the caption of figure 1 into the main text and expanded to better explain the cut-off used (lines 165 – 171). We have also renamed them to “zoonotic potential categories” to avoid implying a direct link to their priority in public health policy.

14. line 163-164: "such that increased similarity to human genomes did not always increase the likelihood of infecting humans" .. this sounds pretty important and interesting. Can you say more here? Do you know what types of similarity do and don't correspond to more likely human infection? Do you know why?

This text has been expanded on lines 247 – 253, with a more detailed discussion added to the supplementary text (S1 Text). However, many of these features are highly correlated in naturally circulating viral genomes, making it difficult to explore general patterns from our data alone. In particular, we were unable to identify clear patterns in terms of which broad classes of features follow or do not follow the expected pattern of genome mimicry, although at least some the apparent “anti-mimicry” appears to be explained simply by trade-offs between correlated features in our data (see supplementary text). We therefore point out the need for direct experimental exploration of these features on lines 438 – 455.

15. lines 180-182. What are these 55 viruses? Sarbecoviruses? Betacoronaviruses? Does this list include both SARS1 and SARS2?

Our analysis included all recognized coronavirus species plus 62 (55 in the previous version of the manuscript) representatives of the species *Severe acute respiratory syndrome-related coronavirus*, which according to the current ICTV taxonomy includes both the original SARS-CoV and SARS-CoV-2. All viruses are named in figure 5B. We have also clarified the sentence in question (lines 329 – 333).

16. line 186, SARS1 or SARS2?

SARS-1 was excluded since it was part of the original training data. We have changed this sentence (line 374 – 376) to emphasise that the models used to make these Sarbecovirus predictions contained no SARS-CoV genomes, as we are testing how the model would have performed had a bat-associated Sarbecovirus been detected before this subgenus was known to infect humans (i.e., before the emergence of SARS-1).

17. Fig S10. Is the x-axis here "CTG codon usage" (i.e. proportion of Leucines coded for by CTG/CUG), or is it some other bias metric?

It is the CTG codon usage bias, using the standard definition of codon usage bias, i.e. the proportion of all Leucines which are encoded by CTG. We have corrected the x-axis label of this figure for clarity (now figure S12).

18. line 317 "class-stratified", i.e. "stratified by virus taxonomic class", yes?

No, stratification was by human-infecting vs. not human-infecting only. Additional stratification by e.g. virus family was not possible because of small sample sizes at this level, which would have resulted in a highly biased, non-random selection of test cases. This has been clarified on lines 570 – 574.
