# Supplementary material for: Identifying and prioritizing potential human-infecting viruses from their genome sequences
Source: PLoS Biol. 2021 Sep 28;19(9):e3001390. doi: 10.1371/journal.pbio.3001390 (PMC8478193; doi:10.1371/journal.pbio.3001390)
Supplement: Attachment — Submitted filename: round3_response_to_reviewers_V2.docx [file pbio.3001390.s017.docx]

# Point-by-point response

## Reviewer 1:

I am satisfied with the changes the authors have made to the manuscript.

## Reviewer 2:

1. The SHAP value is still barely explained or not explained. This made pages 9 and 10 of the new ms very hard to follow. It also made most of Figure 2 very hard to understand. I can't tell if this is my shortcoming in this area, or if this is just an opaque measure that will be hard for all biologists to understand. Is a paragraph on the SHAP value and some equations/methods appropriate here? Other biologists will be reading this.

We have now added an extended explanation at the first mention of this term, including two new citations giving further information (line 197):

**“The relative influence of each genomic feature on prediction outcomes was measured using the SHAP algorithm, which computes the Shapley value for each feature and is increasingly used to improve the interpretability of the decisions made by machine learning models [18]. Shapley values derive from game theory and represent the average marginal contribution of a feature to a prediction across all possible combinations of features [19]. SHAP thus represents complex models as a more interpretable linear combination of values which add up to the final model prediction. As such, SHAP values give a model-agnostic measure of how important features are relative to each other when predicting the human infection-ability of a given virus. Here, high levels of similarity in SHAP values between viruses would indicate that they were predicted to have the same human infection status because of the same patterns in their genomic features [20]. Our analysis therefore asked to what extent such similar uses of the same genomic features followed established taxonomic relationships among viruses.”**

We hope this addresses the reviewer’s concerns and adequately complements existing explanations in the methods section.

Some other points

2. Line 80, exert not assert

Changed.

3. Line 90, "we first build statistical models that assign a probability of zoonotic occurrence"

We agree that this is clearer. The line has been changed to:

**“we first build machine learning models which assign a probability of human infection”**

4. Line 109, I would remove the work "predictably"

Agreed.

5. Line 289, remove 95%

We are referring to an interquantile range, not an interquartile range. While the latter has a fixed meaning and would not require any further qualification, an interquantile range can be between any pair of quantiles – we specifically refer to the range encompassing 95% of the data, i.e. spanning between the 0.025 to 0.975 quantiles.

6. lines 329-332 do not make sense. SARS is the human virus, so you can't call these the 62 human and animal genomes of SARS. Please use individual lineages or sarbecovirus as a classification here.

This has been changed to “sarbecovirus” throughout, but for the purposes of our model it is important that the ICTV currently classifies all of these genomes as the same species^[1]^, as this affects how we generate predictions. The sentence therefore now reads:

**“Specifically, we predicted the zoonotic potential of all currently recognized coronavirus species, along with 62 human and animal-derived *Sarbecovirus* genomes all currently classified by the ICTV as *Severe acute respiratory syndrome-related coronavirus* [23].”**

7. Likewise, "within SARS coronavirus" on line 369 does not make sense. You probably should use lineage or sub-genus here again.

This has been changed to **“among sarbecoviruses”**. The relevant sentence now reads (changes underlined):

**“Among sarbecoviruses, most genomes (85.5%) were classified as having medium zoonotic potential, including the causal agent of the 2003 severe acute respiratory syndrome outbreak (Fig 5B).”**

1. *Coronaviridae* Study Group of the International Committee on Taxonomy of Viruses. The species *Severe acute respiratory syndrome-related coronavirus*: classifying 2019-nCoV and naming it SARS-CoV-2. Nat Microbiol. 2020;5: 536–544. doi:10.1038/s41564-020-0695-z
